# Dynamics of SARS-CoV-2 Spike RBD Protein Mutation and Pathogenicity Consequences in Indonesian Circulating Variants in 2020–2022

**DOI:** 10.3390/genes15111468

**Published:** 2024-11-14

**Authors:** Nabiel Muhammad Haykal, Fadilah Fadilah, Beti Ernawati Dewi, Linda Erlina, Aisyah Fitriannisa Prawiningrum, Badriul Hegar

**Affiliations:** 1 Undergraduate Program of Medicine, Faculty of Medicine, Universitas Indonesia, Jakarta 10430, Indonesia; nabiel.muhammad@ui.ac.id; 2 Department of Medical Chemistry, Faculty of Medicine, Universitas Indonesia, Jakarta 10430, Indonesia; linda.erlina22@ui.ac.id; 3 Bioinformatics Core Facilities, Indonesian Medical and Education Research Institute, Faculty of Medicine, Universitas Indonesia, Jakarta 10430, Indonesia; aisyahfitriannisa@gmail.com; 4 Department of Clinical Microbiology, Faculty of Medicine, Universitas Indonesia, Jakarta 10430, Indonesia; beti.ernawati@ui.ac.id; 5 Department of Child Health, Faculty of Medicine, Universitas Indonesia, Jakarta 10430, Indonesia; badriulh@gmail.com

**Keywords:** variants, pathogenicity, phylogenetic, mutation, RBD, SARS-CoV-2 spike protein

## Abstract

Background: Since the beginning of the coronavirus disease 2019 (COVID-19) outbreak, dynamic mutations in the receptor-binding domain (RBD) in the severe acute respiratory syndrome coronavirus 2 (SARS-CoV-2) spike protein have altered the pathogenicity of the variants of the virus circulating in Indonesia. This research analyzes the mutation trend in various RBD samples from Indonesia published in the Global Initiative on Sharing All Influenza Data (GISAID) database using genomic profiling. Method: Patients in Indonesia infected with SARS-CoV-2, whose samples have been published in genomic databases, were selected for this research. The collected data were processed for analysis following several bioinformatics protocols: visualization into phylogenetic trees, 3D rendering, and the assessment of mutational impact. Results: In Indonesia, there are 25 unique SARS-CoV-2 clades and 318 unique SARS-CoV-2 RBD mutations from the earliest COVID-19 sample to samples collected in 2022, with T478K being the most prevalent RBD mutation and 22B being the most abundant clade. The Omicron variant has a lower docking score, higher protein destabilization, and higher K_D_ than the Delta variant and the original virus. Conclusions: The study findings reveal a decreasing trend in virus pathogenicity as a potential trade-off to increase transmissibility via mutations in RBD over the years.

## 1. Introduction

On 11 March 2020, the World Health Organization (WHO) declared coronavirus disease 2019 (COVID-19) a pandemic threat; COVID-19 is an infectious disease caused by severe acute respiratory syndrome coronavirus 2 (SARS-CoV-2) that has had a devastating impact on the world [1]. Based on WHO data, as of 31 December 2022, there had been a total of 729,470,750 confirmed COVID-19 cases and 6,718,860 reported deaths globally [2]. In Indonesia, there were 6,719,815 confirmed COVID-19 cases and 160,612 confirmed deaths, based on data from the WHO and the Indonesian Ministry of Health [3,4]. This might be due to the severity of COVID-19 and the highly contagious nature of the SARS-CoV-2 virus, which might explain the high rates of infection and death. The transmission of the SARS-CoV-2 virus from infected individuals to a new host occurs when droplets from the infected individual enter the nose or mouth of a healthy individual. Thus, healthy individuals can become infected with SARS-CoV-2 through everyday incidents such as sneezing, coughing, and talking, without noticing [5].

Many countries implemented many preventive measures to slow the spread of the SARS-CoV-2 infection, including social distancing, the regular washing of hands, curfews, limitations to the size of gatherings in public places, and vaccination [5]. In particular, vaccination is considered the most viable solution in humanity’s efforts to fight COVID-19, with many countries collaborating and contributing to the creation of vaccines to help protect humans from the virus [6,7]. Vaccines prompt the immune system of the recipient to fight specific pathogenic organisms. Consequently, in the event of an infection, the human body will recognize the virus against which it has been vaccinated and the immune system will already have been primed to fight off the actual virus [8].

However, being vaccinated against SARS-CoV-2 does not mean that one is invulnerable to the virus. There had been approximately 13,008,560,983 COVID-19 vaccine doses administered as of 12 December 2022; nonetheless, there were still several new COVID-19 cases daily [5]. Remarkably, this surge in SARS-CoV-2 infection rates was most likely due to gene mutations that alter the characteristics of the virus, contributing to the growing number of variants emerging globally. This phenomenon occurs due to the unstable nature of the virus, which tends to mutate continually. SARS-CoV-2 mutations can occur when the virus is transmitted and infects a new host, as the virus adapts to the environment. The more instances of transmission there are, the greater the probability of the virus mutating. This explains why COVID-19 rapidly evolves into many variants [2].

Because of these emerging mutations, researchers have been trying to create a nomenclature system for SARS-CoV-2 strains for clear delineation [9]. SARS-CoV-2 viruses are categorized in several different ways, with the applicable classification depending on how SARS-CoV-2 is transmitted. SARS-CoV-2 lineages are frequently brought up in scientific discourse; the Phylogenetic Assignment of Named Global Outbreak (PANGO) nomenclature system is the most predominantly employed lineage categorization scheme. The WHO may use Greek letters to classify lineages or—in a more general sense—clusters of similar lineages [10]. Similarly, Nextstrain classifies SARS-CoV-2 variants into 14 primary clades, including 19A, 19B, and 20A–20L [11]. Hence, researchers can explain the shared and distinguishing characteristics between strains using these classification techniques. Numerous SARS-CoV-2 variants have been identified in Indonesia, including the B.1.466.2 lineage, which originated in Indonesia, and the Alpha, Beta, Delta, and Omicron variants. These variants have been linked to surges in the number of COVID-19 cases confirmed worldwide. Notably, genomic surveillance has revealed that a sizable fraction of SARS-CoV-2 strains in Indonesia had already switched from the Wuhan strain to various other variants—predominantly the B.1.466.2 variant—between the end of 2020 and the beginning of 2021 [12,13]. Regrettably, there is a dearth of studies that elucidate the dynamics of SARS-CoV-2 variants in Indonesia using Nexclade labeling, which is analyzed in this study.

To prevent an upsurge in viral infections in the near future, there is a dire need to develop new COVID-19 prevention countermeasures while improving the available countermeasures [14,15]. The viral spike protein is crucial in angiotensin-converting enzyme 2 (ACE2) receptor binding and subsequent membrane fusion and is thus critical in research on COVID-19 treatment methods and immunological development. Therefore, mutations in the SARS-CoV-2 spike protein—especially the receptor-binding domain (RBD)—must be thoroughly researched. However, to date, there is a dearth of data on how the RBD mutation has developed over time in Indonesia. To obtain an understanding of such mutation patterns in Indonesia, it is necessary to possess knowledge of the relative mutation frequencies across the country and to facilitate the prediction of the emergence of new variants—which can be achieved using genomic profiling [16]. Therefore, this study provides information on the mutation trends of the SARS-CoV-2 spike (*S*) *gene*—especially for RBD mutations—to aid in the development of new measures or the improvement of the most effective existing measures against potential emerging variants.

## 2. Methods

### 2.1. Indonesian SARS-CoV-2 Genome Data Retrieval

All sequenced SARS-CoV-2 genome data are stored in the Global Initiative on Sharing All Influenza Data (GISAID) database at https://gisaid.org/ (EPI_SET ID: EPI_SET_230913pu) (accessed on 12 February 2023) [17]. Data on COVID-19 from all over Indonesia spanning 2020 to 2022 and stored in the EpiCoV GISAID database were included in this study [17]. The *Asia*/*Indonesia* location criterion was used to search for genomic sequence samples from Indonesia, with complete and human hosts applied as a filter, yielding 46,149 sequences (EPI_SET ID: EPI_SET_230913qz). The sequences were further analyzed using Nextstrain (https://clades.nextstrain.org/) (accessed on 12 February 2023) [18], and sequences with an overall quality control (QC) that was determined to be bad (3218), mediocre (3759), or blank (2) were discarded, resulting in 39,170 sequences.

### 2.2. Phylogenetic Tree Reconstruction

To determine the origins and groupings of the SARS-CoV-2 variants, the phylogenetic tree was reconstructed based on the aligned sequence using Auspice (https://auspice.us/) (accessed on 12 February 2023).

### 2.3. Three-Dimensional Rendering of SARS-CoV-2 Protein and Assessment of Mutation Impact

After reconstructing the phylogenetic tree, a 3D structural visualization of SARS-CoV-2 was rendered using the University of California, San Francisco (UCSF) Chimera version 1.6.1 (University of California, San Francisco, CA, USA) [19] software to analyze the structural effect of RBD mutations. Subsequently, a SARS-CoV-2 model was docked using HDOCK web server (School of Physics, Huazhong University of Science and Technology, Wuhan, China) [20], and PRODIGY web server for protein-protein binding affinity prediction (University of Utrecht, Utrecht, Netherlands) [21] was then used to assess the dissociation constant of the SARS-CoV-2 model. Finally, the effects of the RBD mutations on protein stability were assessed using DynaMut2 web-server (the latest version of DynaMut) (University of Melbourne, Melbourne, Australia) [22].

## 3. Results

### 3.1. Identification of Clades and Phylogenetic Trees

**Figure 1:**
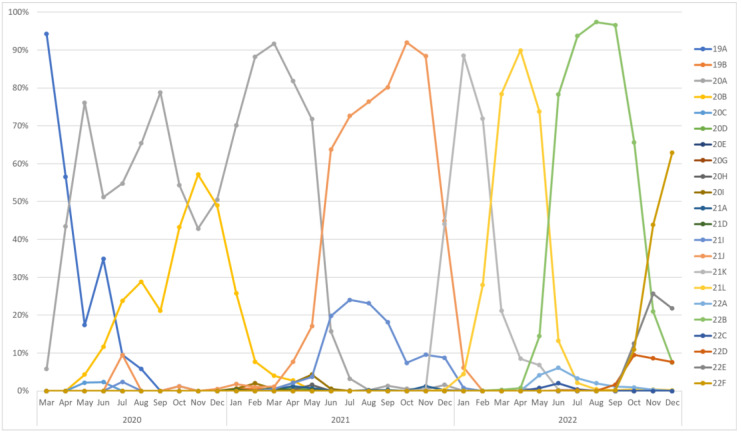
SARS-CoV-2 NextStrain clades observed in Indonesia. This figure shows Indonesia’s 25 unique severe acute respiratory syndrome coronavirus 2 (SARS-CoV-2) clades’ growth and fluctuations from 2020 to 2022. In 2020, the 19A clade (the original Wuhan strain) was most frequent from May to July, then gradually declined and was replaced by the 20A clade, which became dominant until the end of the year. In 2021, 20A was initially dominant but was overtaken by the 21J clade (a Delta variant subclade) in the year’s second half. By December 2021, the 21K clade (Omicron or BA.1) surged in prevalence. In 2022, 21K peaked in January but declined by June, replaced by the 22B clade (Omicron or BA.5). Later in 2022, 22F (Omicron or XBB) and 22E (Omicron or BQ.1) became the most observed clades. Overall, 22B was the most frequently observed clade, followed by 21K, 21J, 22F, and 21L.

Nextstrain analysis revealed 25 unique SARS-CoV-2 clades; the growth and fluctuations in the prevalence of these clades are outlined in Figure 1. From May to July 2020, 19A was the most prevalent clade in Indonesia; 19A is the original SARS-CoV-2 strain (i.e., the Wuhan strain) discovered during the initial outbreak of the pandemic [23,24]. From May to July 2020 in Indonesia, the prevalence of 19A declined gradually, and it was then replaced in July 2020 by 20A, the prevalence of which inclined substantially toward December 2020. Then, 20A, the dominant SARS-CoV-2 clade in the predominant European outbreak in early 2020, evolved from 19A, its progenitor. In 2021, 20A was initially the most dominant clade globally, until it began exhibiting a declining trend in May and was replaced by 21J (a subclade of the Delta variant) as the most abundant clade in Indonesia in the second half of 2021 [25]. Remarkably, there was a spurt in 21K (Omicron or BA.1) growth, which was almost as abundant as 21J in December 2021; 21K was first reported in South Africa in November 2021. In addition, during the first wave of COVID-19 in Indonesia, 20A was the most prevalent clade [26], and during the second wave, 21J, 21I, and 20a were reported as the most prevalent clades in Indonesia.

There was a significant increase in the prevalence of 21K in 2022, especially in January. However, this prevalence of 21K began declining until June and was then replaced by 22B (Omicron or BA.5). The 22B clade possibly emerged in the initial months of 2022 in South Africa. Similarly, 22B began diminishing in prevalence after July, and was then replaced by 22F (Omicron or XBB) and 22E (Omicron or BQ.1), two of the most frequently reported clades in Indonesia. These two clades, 22E and 22F, possibly emerged in Western or Central Africa, while 22E and 22F possibly emerged in South Asia in the middle of 2022. In sum, the most frequently reported clade was 22B (30.68%), followed by 21K (17.92%), 21J (13.65%), 22F (11.74%), and 21L (7.32%). In contrast, the other clades were much less predominant, accounting for only less than a tenth of the total sequences. Remarkably, the 21K and 21L clades were predominant during the third wave.

After the Nextstrain analysis, Auspice tools were used to create a phylogenetic tree (Figure S1). During the first wave of COVID-19, the SARS-CoV-2 clades had already exhibited diversity, with 21J being the most divergent clade in Indonesia, followed by the 20A clade, which was the most prevalent clade during this wave (Figure S2). Notably, the 21J clade diverged from 21A, which evolved from 20A. During the second wave, 21J was the most divergent clade, followed by 20D and 20A (Figure S3). In addition, the 21J and 21I clades diverged from 21A, while 20D diverged from 20B. Compared to the previous wave, the clades showed more diversity, which correlated with increased virus fitness and pathogenicity consequences. During the last wave, 21L was the most divergent clade, followed by 21K, which diverged from 21M (Figure S4). This high divergence during the third wave vis à vis the previous waves may be correlated with the number of reported cases.

### 3.2. Identification of SARS-CoV-2 RBD Mutation in Indonesia

**Figure 2:**
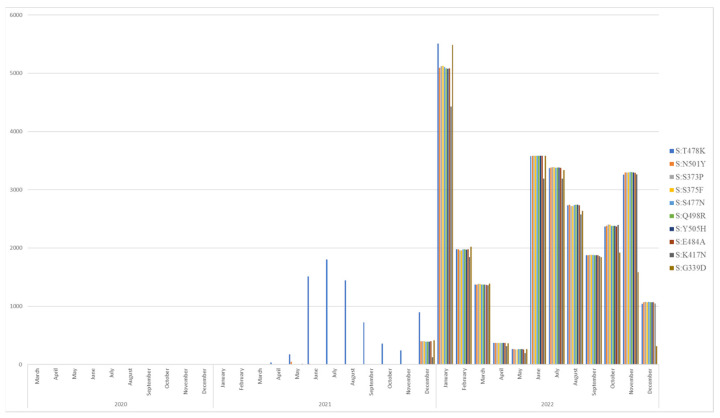
RBD mutations in Indonesia and their growth trends. This figure shows how receptor-binding domain (RBD) mutations in Indonesia evolved from 2020 to 2022. In March 2020, we started with just one unique mutation, A352S. Over time, the number of unique mutations grew significantly, reaching 318 by December 2022. From January to May 2021, N439K stayed dominant but was gradually overtaken by L452R, which peaked in July before declining. Between November and December 2021, 18 different RBD mutations rose significantly, including T478K and L452R. By 2022, the mutation landscape had become quite diverse, with 15 prominent RBD mutations occurring over 19,000 times, including T478K, S373P, S375F, S477N, N501Y, Q498R, Y505H, E484A, K417N, G339D, N440K, D405N, T376A, S371F, and R408S. Meanwhile, Q493R, G446S, G496S, S371L, and R346K started to decrease during this year.

The mutations in the sequences were analyzed using the online Nextstrain tool, with a focus on the *S genes* and a range of 333–527 bases, to detect RBD mutations. We found that there have been 318 unique SARS-CoV-2 S protein RBD mutations in Indonesia over the years, with a total of 484,199 mutations detected in 39,170 sequences. The fluctuations and occurrence rates of the top 10 RBD mutations in Indonesia spanning from 2020 to 2022 are presented in Figure 2 and Table 1. The number of RBD mutations has substantially increased since the outset of the pandemic, from only one unique RBD mutation reported in March 2020 in Indonesia to 318 unique mutations detected in December 2022 samples. The A352S mutation was the first RBD mutation discovered, in March 2020 samples. Subsequently, more RBD mutations emerged, peaking in December 2020, with a total of 39 RBD mutations detected. Notably, N439K, which accounted for 45.68% of all RBD mutations in 2020, was also the most prevalent mutation that year, emerging in November and peaking in December.

From January to May 2021, N439K was the most prevalent RBD mutation. However, after May 2021, L452R replaced N439K as the most prevalent RBD mutation. The prevalence of L452R surged significantly in May 2021, eventually peaking in July and subsequently declining until November 2021. Interestingly, from November to December 2021, there was a substantial rise in the prevalence of 18 RBD mutations, including T478K, L452R, N439K, N501Y, G339D, E484A, S373P, S375F, S371L, S477N, Q498R, Q493R, Y505H, G496S, R346K, K417N, G446S, and N440K. Remarkably, the prominent RBD mutations that emerged in 2021 were T478K and L452R, which accounted for 37.57% and 35.47% of all SARS-CoV-2 RBD mutations, respectively, while the others accounted for less than 10%. In 2022, the RBD mutations were quite divergent: there were 15 prominent RBD mutations, with a total count of more than 19,000 mutations throughout 2022, which include T478K, S373P, S375F, S477N, N501Y, Q498R, Y505H, E484A, K417N, G339D, N440K, D405N, T376A, S371F, and R408S. Furthermore, during 2022, Q493R, G446S, G496S, S371L, and R346K gradually began to decline in prevalence.

### 3.3. Total Deaths and Confirmed COVID-19 Cases in Indonesia

**Figure 3:**
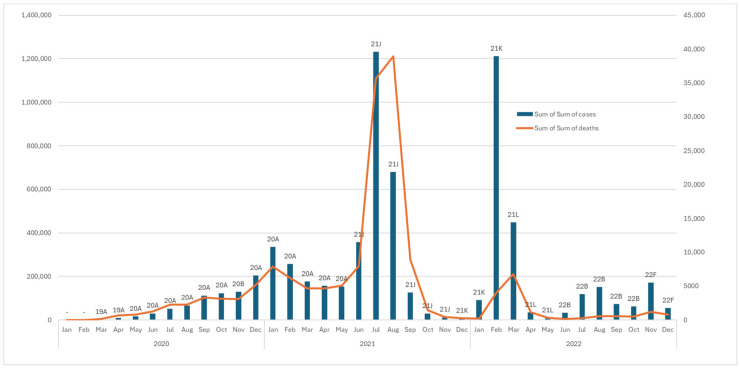
Graph showing COVID-19 cases and deaths, with the most frequent clades circulating in Indonesia. This graph shows the trends in coronavirus disease 2019 (COVID-19) cases and deaths in Indonesia, highlighting the month’s most common viral clades. As of 31 May 2023, Indonesia had reported 6,807,513 confirmed cases and 161,771 deaths. The highest peaks in new cases were in July 2021 (1,231,386 cases) and February 2022 (1,211,078 cases). The most deaths occurred in July 2021 (38,904 deaths) and August 2021 (35,628 deaths), predominantly of the 21J clade.

Indonesia had recorded 6,807,513 confirmed COVID-19 cases and 161,771 deaths as of 31 May 2023. The highest number of fresh confirmed cases was recorded in July 2021, with a total of 1,231,386 cases, predominantly of the 21J clade, followed by February 2022, with 1,211,078 cases, predominantly of the 21K clade. Regarding the death toll, the highest number of deaths occurred in 2021—specifically in July (38,904) and June (35,628), predominantly of the 21J clade [4]. A graph tracing the number of deaths and COVID-19 cases, with the most prevalent clade each month, is presented in Figure 3.

### 3.4. Three-Dimensional Modeling of SARS-CoV-2

**Figure 4:**
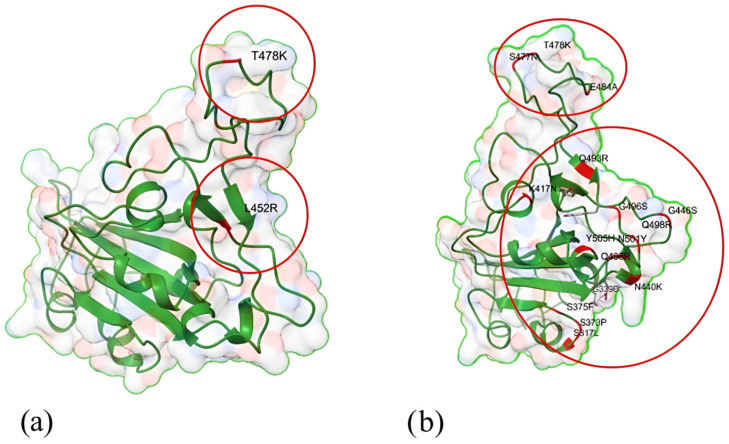
Three-dimensional modeling of SARS-CoV-2 RBD mutations. (**a**) Delta variant RBD: this 3D model shows the Delta variant’s receptor-binding domain (RBD) mutations. The L452R mutation changes the structure from two coils to two sheets in certain regions, while the T478K mutation alters the coiled structure into a turn type, affecting nearby areas. These changes disrupt important bonds, impacting the overall structure. (**b**) Omicron variant RBD: this model illustrates the Omicron variant’s RBD mutations. Although the overall structure is similar to the original strain, the helix has a slight shift. Mutations at various positions lead to significant structural changes, including shifts from polar to nonpolar residues and additions of amine groups. These mutations impact the binding process of the SARS-CoV-2 RBD to ACE2 receptors and interact with antibodies.

A 3D model of the SARS-CoV-2 RBD mutation was rendered using the UCSF Chimera application utilizing protein data (PDB ID: 6M0J) in the Protein Data Bank [27]. Subsequently, the RBD structure was mutated in UCSF Chimera to incorporate the RBD mutations detected in the Omicron and Delta variants. Visualizations of the Delta and Omicron variants are presented in Figure 4. Each mutation in the corresponding residue is labeled in red for easy identification.

In the Delta variant, the L452R substitution changed the two-coil structure in the Wuhan strain protein to a two-sheet structure in the Y449 and N450 regions, as well as in F456. The T478 residue is critical to the coiled-coil structure of the S protein, but the T478K mutation transforms it into a turn-type conformation, which also alters the S477 region. Because of the L452R mutation, only pair-based interactions are possible for the cross-linked hydrophobic connection between L452 and Y351 [28]. Leucine has been substituted in the 452 regions, resulting in an alteration in structure brought on by the addition of amine groups. This substitution destroyed two van der Waals bonds between the L452 residue and the S349 and Q493 regions, as well as a single carbonyl bond connecting L452 to the Q493 residue. Furthermore, the T478K substitution contributes to structural alteration owing to the addition of an amine group and the substitution of threonine with the lysine amino acid. This destroys the van der Waals bond linking T478 with N487 and the two polar bonds linking T478 with the F486 and N487 residues (Figure 4a) [28,29].

Excluding a slight shift in a short helix located at positions 365–371 in the Omicron variant, the general structure of the RBD has not been altered much from the Omicron and G614 trimmers. However, numerous mutations have altered the receptor-binding motif (RBM) surface, which is exposed by the closed conformation of the S protein. The RBM surface comprises the ACE2-binding interface and numerous RBD-1 and RBD-2 antibody epitopes [30]. A carboxylic group was added at position 339, substituting glycine with aspartic acid and thus causing structural alteration. In addition, serine at the 371, 373, and 375 residues was substituted with leucine, proline, and phenylalanine, resulting in polar to nonpolar alterations in the structure. Furthermore, serine was substituted with glycine at positions 446 and 496. Another significant structural modification was detected at residue 484, owing to Alanine substitution with glutamic acid and the resulting carboxyl group loss and methyl group insertion [29].

At residues 493 and 498, glutamine was substituted with arginine, resulting in modifications to the structure, with amine groups added and a hydroxyl group removed. At residue 440, the substitution of amino acid from polar asparagine with positively charged lysine amino acid resulted in a critical structural alteration owing to the elimination of a keto group and the insertion of amine groups. In addition, a substitution in S373P may have stiffened the local polypeptide chain, forcing the helix constructed from sequences 365–371 to shift inward. This, in turn, resulted in a significant N-linked glycan rotation at N343 and may have been caused by the local polypeptide chain (Figure 4b) [29].

### 3.5. RBD and ACE2 Molecular Docking and Dissociation Constant

The HDOCK tool was used to dock the RBD of the wild-type, Delta, and Omicron variants against the native ACE2 model [PDB ID: 1R42] to assess the efficiency of the binding between the RBD and ACE2 [20]. Based on the prediction results (Table 2), we found that the Omicron RBD has the lowest docking score (−295.20 kcal/mol), followed by the Delta RBD (−265.72 kcal/mol), while the RBD of the wild-type variant (−257.03 kcal/mol) had the highest docking score among the lineages.

The PROtein binDIng enerGY prediction (PRODIGY) tool was used to assess the dissociation constant of the RBD in wild-type, Omicron, and Delta variants to determine the biological macromolecule interaction strength [21]. Based on the results (Table 2), we found that the RBD of the wild-type variant has a K_D_ of 1.9 nM, the RBD of the Delta variant has a K_D_ of 1.8 nM, and the RBD of Omicron has a K_D_ of 3.6 nM.

### 3.6. Impact of Mutations on RBD Protein Stabilization in Omicron and Delta Variants

The impact of the mutations reported in the Omicron and Delta variants on RBD protein stability was assessed using DynaMut2 and the multiple-mutation method. Mutants with predicted Gibbs free energy (G) values lower than zero were categorized as destabilizing, while those with G values greater than zero were categorized as stabilizing. Notably, kcal/mol was used as the unit of protein stability. Two RBD mutations were detected in the Delta variant: L452R and T478K. L452R (−0.53 kcal/mol) was predicted to be destabilizing, while T478K (0.06 kcal/mol) was predicted to be stabilizing (Table 3). In the Omicron variant, 15 RBD mutations were detected, including Q493K, N501Y, S375F, S371L, G496S, S477N, K417N, G446S, Q498R, N440K, E484A, G339D, Y505H, and S373P. Five mutations were predicted to be stabilizing: E484A (0.12 kcal/mol), N440K (0.32 kcal/mol), S373P (0.08 kcal/mol), S477N (0.12 kcal/mol), and T478K (0.06 kcal/mol). The remaining 10 RBD mutations were predicted to be destabilizing: Q493K (−0.47 kcal/mol), N501Y (−0.62 kcal/mol), S375F (−0.65 kcal/mol), S371L (−0.31 kcal/mol), G496S (−0.7 kcal/mol), K417N (−1.51 kcal/mol), G446S (−0.8 kcal/mol), Q498R (−0.31 kcal/mol), G339D (−0.28 kcal/mol), and Y505H (−0.98 kcal/mol).

## 4. Discussion

The samples used in this research span from 2020 to 2022, as this period saw significant increases in new COVID-19 cases and mortality (Figure 3), which may be linked to the pathogenicity and transmissibility of the virus. Samples from 2023 were not included in this study because SARS-CoV-2 was less prevalent in 2023 than during the preceding years and has slowly been diminishing in Indonesia in terms of the number of new cases and mortality rate, based on WHO data. Furthermore, the GISAID sample collection for the year 2023 is not yet complete; therefore, there could have been errors in our analysis if 2023 had been included in this study. The Delta and Omicron variants were selected as the focus to explore the impact of mutations on pathogenicity, as these two variants exhibited high transmissibility from 2020 to 2022. However, the Omicron variant exhibited a lower mortality rate than the Delta variant [30,31,32].

### 4.1. Association Between Mutation and New Confirmed COVID-19 Cases and Mortality in Indonesia

During the first wave of COVID-19 (November 2020 to January 2021), a considerable number of new cases were reported in Indonesia. The clade 20A was the most prevalent during this period, followed by 20B. According to our analysis, the most prevalent 20A SAR-CoV-2 lineage was B.1.466.2, which originated in Indonesia [33]. This variant possesses a unique RBD mutation, N439K, which has an occurrence rate of over 98% [34]. Remarkably, the N439K mutation was a frequently detected RBD mutation throughout the year, which aligns with 20A being the dominant clade during this period. SARS-CoV-2 with this type of mutation has a higher hACE2 affinity than the wild-type strain, and the mutation reduces monoclonal and polyclonal antibody neutralization activity, thus facilitating immune escape; in contrast, the wild-type strain has a higher viral infectivity [35,36]. The clade 20B evolved from 20A, bearing N203K, N204R, and ORF1450N mutations. During the first wave of COVID-19, the two most frequently reported lineages from 20B were B.1.1.398 (an Indonesian-associated variant) and B.1.1.53.

During the second wave of COVID-19 (May to September 2021), three of the most prevalent clades reported were 21J, 21I, and 20A. However, the number of 20A variants found in the samples analyzed in this study began declining after June 2021—although this clade was the most prevalent in May and June 2021—and was eventually replaced as the most prevalent clade by 21J and 21I. Notably, there were a high number of COVID-19 deaths in June (35,628) and July (38,904) 2021 during the second wave. The lineage of 20A during this period was still the same as during the previous wave, which was B.1.466.2. Furthermore, 21J (a subclade of the Delta variant) has two RBD mutations: L452R and T478K. The RBD L452R mutation is linked to immune escape and makes the virus more infectious and fusogenic, encouraging viral reproduction and boosting the transmissibility and pathogenicity of the virus. The T478K mutation has a similar effect on SARS-CoV-2 to L452R; both mutations contribute to immune escape, neutralizing antibody resistance, and improved stabilization between hACE2 and RBD [37]. Clade 21I (a subclade of the Delta variant) is similar to 21J, and 21I possesses every 21A mutation along with additional mutations in *ORF1a* and the *S genes*.

Finally, during the third wave of COVID-19, we found that the most prevalent clades were 21K and then 21L. The clade 21K (a subclade of the Omicron variant) has numerous *S genes* mutations in the N-terminal and the RBD that are crucial in antibody recognition and binding to hACE2; these mutations are S:E484A, S:Q498R, and S:N501Y. The mutation at S:E484A has been linked to immune escape, and in vitro evolution experiments have revealed that the mutations located at S:Q498R and S:N501Y—which are RBD mutations—dramatically boost hACE2 binding affinity when acting jointly. Notably, during this wave, many unique RBD mutations—which are found mostly in clade 21K—were prevalent in SARS-CoV-2 viruses in Indonesia: S:S477N, S:T478K, S:Q498R, S:N501Y, S:E484A, S:Y505H, S:S373P, S:Q493R, S:S375F, S:K417N, S:N440K, S: G496S, S: S371L, and S: G446S.

Although the number of new COVID-19 cases during the third wave was almost as high as during the second wave and there were many RBD mutations reported during the third wave, there was no significant increase in mortality due to COVID-19. This might be explained by studies that report that the Omicron variant exhibits greater infectivity than the Delta variant but with a lower case fatality globally, which is primarily attributable to its pathogenicity being reduced by mutation [38]. However, this may also be attributable to the higher vaccination coverage compared to the coverage during the previous wave, which also lowered the fatality rate in COVID-19 patients.

### 4.2. Effect of Mutations on the Pathogenicity of the Omicron and Delta Variants

S-RBD binding to hACE2 is a crucial process in the infectivity and transmissibility of SARS-CoV-2. In addition, it is surmised that a strong RBD–hACE2 interaction may be linked to the hyper-transmissibility of the Omicron variant [39]. The Omicron variant is considerably divergent compared to the wild-type Wuhan strain and the Delta variant, which has only two RBD mutations. In contrast, the Omicron variant carries approximately 15 RBD mutations, which can be linked to a high ACE2-binding affinity and an improved likelihood of evading the host’s immune system. This aligns with our study results, in which the Omicron variant has a lower binding energy score than the Delta and wild-type strains (Table 3). Furthermore, these results align with those of Barh et al. (2023), who found the Omicron variant to have a lower docking score than the wild-type and Delta variants [39]. This indicates that the Omicron variant has the highest ACE2 binding affinity among the SARS-CoV-2 strains, contributing to its higher transmissibility [39,40].

However, based on the PRODIGY results (Table 2), we found that the Omicron variant has a higher dissociation constant (K_D_) than the wild-type variant, while the Delta variant has a lower K_D_, which indicates that the Delta variant has a higher binding affinity than the Omicron variant [41]. This finding conflicts with that of Nguyen et al. (2022), who reported a lower dissociation constant for the Omicron variant than for the wild-type variant [41]. Nonetheless, there are many studies with conflicting research findings on this subject, with several studies reporting higher binding affinities for the Omicron RBD than the other variant, while others, such as the 2023 study by Barh et al., report lower binding affinities [39].

Although the RBD of Omicron having substantial binding affinity for hACE2 may result in high viral transmissibility, this affinity may not be sufficient for systemic infection because of the unstable state of the Omicron S protein, which can cause severe COVID-19 [38,39]. We found that Omicron strains possess ten destabilizing RBD mutations, Q493K (−0.47), N501Y (−0.62), S375F (−0.65), S371L (−0.31), G496S (−0.7), K417N (−1.51), G446S (−0.8), Q498R (−0.31), G339D (−0.28), and Y505H (−0.98), and only five stabilizing RBD mutations, E484A (0.12), N440K (0.32), S373P (0.08), S477N (0.12), and T478K (0.06), with the accumulation of −5.97 kcal/mol. In contrast, the Delta strain has only one stabilizing RBD mutation, T478K (0.06), and one destabilizing RBD mutation, L452R (−0.53), with the accumulation of −0.47 kcal/mol. Therefore, it has been proposed that the Omicron RBD is less stable than the RBDs of other strains as a result of amino acid substitutions, which reduces the probability of its host developing COVID-19 [40,42]. Notably, the reduced disease severity and fewer fatalities of COVID-19 caused by the Omicron strain are linked to the attenuated replication ability of this strain of the virus [39,43]. Similarly, destabilizing mutations are typically harmful and do not provide a fitness advantage; they cause a decline in virus production and infectivity [44].

The findings of a 2022 study by Shuai et al. indicate that in exchange for the ability to avoid neutralization by antibodies, the replication fitness of the Omicron variant may have been severely impaired [43]. Prior to the emergence of the Omicron variant, SARS-CoV-2 variants were known to have undergone changes in the spike proteins that affected the effectiveness of viral transmission and easily permeated the human respiratory tract [43]. This may be due to SARS-CoV-2 adapting in order to escape vaccines and antibodies by accumulating mutations beneficial to transmissibility [45]. Furthermore, Shuai et al. report a decreasing pathogenicity trend for SARS-CoV-2 variants based on a comparison of their pathogenicity in transgenic mouse models [43]. In addition, a 2023 study conducted by Barh et al. found a similar trend in the pathogenicity of SARS-CoV-2 variants via assessments using the Mutation Parser 3 (MP3) tool and by assessing the diminished ability of the Omicron variant to induce pro-inflammatory cytokines. The findings of these studies may explain the declining pathogenicity of Omicron strains [36]. However, our study has certain limitations, as the patients whose samples were used may have contracted COVID-19 due to geographical and host-related factors that increase the transmissibility and pathogenicity of SARS-CoV-2, and there is also a lack of metadata on GISAID samples from Indonesia.

## 5. Conclusions

Based on the findings of this study, it can be proposed that there are 25 unique SARS-CoV-2 clades and 318 unique SARS-CoV-2 RBDs in Indonesia, spanning from the earliest samples to samples collected in 2022. T478K was the most prevalent RBD mutation, and 22B was the most abundant clade reported in Indonesia during the study period. In addition, the Omicron variant has a lower docking score, higher protein destabilization, and a higher K_D_ than the Delta and wild-type strains. These indicate a decreasing trend in the pathogenicity of the virus, potentially as a trade-off for increased transmissibility via RBD mutations over the years. In this study, we did not focus on specific regions of the genome but analyzed the entire sequence from samples submitted in Indonesia. Consequently, the dynamics of the mutations and clades are generalized and may not explain how the viruses are transmitted in various regions of Indonesia, which may have different clades and RBD mutations in different parts of each region. Therefore, it is better to focus on an area by considering regional geographic factors to determine how clades and RBD mutations develop.

## Figures and Tables

**Table 1:** Top ten most frequent RBD mutations in Indonesia.

Nucleotide Substitution	Amino Acid Change	Occurrence Rate	Mutation Type
C22995A	T478K	7.22%	Non-synonymous
A23063T	N501Y	5.76%	Non-synonymous
T22679C	S373P	5.75%	Non-synonymous
C22686T	S375F	5.75%	Non-synonymous
G22992A	S477N	5.75%	Non-synonymous
A23055G	Q498R	5.74%	Non-synonymous
T23075C	Y505H	5.74%	Non-synonymous
A23013C	E484A	5.73%	Non-synonymous
G22813T	K417N	5.33%	Non-synonymous
G22578A	G339D	5.20%	Non-synonymous

**Table 2:** Docking score and dissociation constant of wild-type, Omicron, and Delta variants against native hACE2 using HDOCK and PRODIGY.

RBD	Docking Score (Kcal/mol)	Dissociation Constant (K_D_)
Wild-type	−257.03	1.9 nM
Delta	−265.72	1.8 nM
Omicron	−295.20	3.6 nM

**Table 3:** Predicted Gibbs free energies of Omicron and Delta variant RBD mutations using DynaMut2.

Mutation	Predicted ΔΔG (Kcal/mol)	Outcome
N440K	0.32	Stabilizing
S477N	0.12	Stabilizing
E484A	0.12	Stabilizing
S373P	0.08	Stabilizing
T478K	0.06	Stabilizing
G339D	−0.28	Destabilizing
S371L	−0.31	Destabilizing
Q498R	−0.31	Destabilizing
Q493K	−0.47	Destabilizing
L452R	−0.53	Destabilizing
N501Y	−0.62	Destabilizing
S375F	−0.65	Destabilizing
G496S	−0.7	Destabilizing
G446S	−0.84	Destabilizing
Y505H	−0.98	Destabilizing
K417N	−1.51	Destabilizing

## Data Availability

All sequenced genome data can be publicly accessed in the GISAID database (EPI_SET ID: EPI_SET_230913pu).

## Supplementary Materials

The following supporting information can be downloaded at: https://www.mdpi.com/article/10.3390/genes15111468/s1, Figure S1: Phylogenetic tree model of SARS-CoV-2 circulating in Indonesia created using NextStrain; Figure S2: Phylogenetic tree model of SARS-CoV-2 circulating in Indonesia during the first wave created using NextStrain; Figure S3: Phylogenetic tree model of SARS-CoV-2 circulating in Indonesia during the second wave created using NextStrain; Figure S4: Phylogenetic tree model of SARS-CoV-2 circulating in Indonesia during the third wave created using NextStrain.

## References

[1] Centers for Disease and Control and Prevention (2020). CDC Museum COVID-19 Timeline|David J. Sencer CDC Museum|CDC. https://www.cdc.gov/museum/timeline/covid19.html.

[2] World Health Organization (2020). Coronavirus Disease (COVID-19): Virus Evolution.

[3] World Health Organization Number of COVID-19 Cases Reported to WHO. WHO Data. https://data.who.int/dashboards/covid19/cases?n=c.

[4] Ministry of Health of Republic Indonesia (2020). Dashboard of COVID19 Situation.

[5] World Health Organization (2020). Coronavirus Disease (COVID19).

[6] World Health Organization (2020). 172 Countries and Multiple Candidate Vaccines Engaged in COVID-19 Vaccine Global Access Facility.

[7] Kasai T. (2021). COVID-19 Vaccines Offer Hope, Other Prevention Measures Must Continue.

[8] World Health Organization (2020). How Do Vaccines Work.

[9] Khan T., Jamal S.M. (2021). SARS-CoV-2 Nomenclature: Viruses, Variants and Vaccines Need a Standardized Naming System. Future Virol..

[10] Centers for Disease Control and Prevention (2020). COVID Data Tracker: Variant-Proportions.

[11] Flores-Vega V.R., Monroy-Molina J.V., Jiménez-Hernández L.E., Torres A.G., Santos-Preciado J.I., Rosales-Reyes R. (2022). SARS-CoV-2: Evolution and Emergence of New Viral Variants. Viruses.

[12] Tenda E.D., Asaf M.M., Pradipta A., Kumaheri M.A., Susanto A.P. (2021). The COVID-19 surge in Indonesia: What we learned and what to expect. Breathe.

[13] Wijayanti N., Gazali F.M., Supriyati E., Hakim M.S., Arguni E., Daniwijaya M.E.W., Nuryastuti T., Nuhamunada M., Nabilla R., Haryana S.M. (2022). Evolutionary dynamics of SARS-CoV-2 circulating in Yogyakarta and Central Java, Indonesia: Sequence analysis covering furin cleavage site (FCS) region of the spike protein. Int. Microbiol..

[14] Chakraborty C., Sharma A.R., Bhattacharya M., Lee S.-S. (2022). A Detailed Overview of Immune Escape, Antibody Escape, Partial Vaccine Escape of SARS-CoV-2 and Their Emerging Variants with Escape Mutations. Front. Immunol..

[15] Maragakis L. (2021). Coronavirus Second Wave, Third Wave and Beyond: What Causes a COVID Surge.

[16] Moni M.A., Quinn J.M.W., Sinmaz N., Summers M.A. (2021). Gene expression profiling of SARS-CoV-2 infections reveal distinct primary lung cell and systemic immune infection responses that identify pathways relevant in COVID-19 disease. Brief. Bioinform..

[17] Shu Y., McCauley J. (2017). GISAID: Global initiative on sharing all influenza data—From vision to reality. Eurosurveillance.

[18] Aksamentov I., Roemer C., Hodcroft E.B., Neher R.A. (2021). Nextclade: Clade Assignment, Mutation Calling and Quality Control for Viral Genomes. J. Open Source Softw..

[19] Pettersen E.F., Goddard T.D., Huang C.C., Couch G.S., Greenblatt D.M., Meng E.C., Ferrin T.E. (2004). UCSF Chimera—A visualization system for exploratory research and analysis. J. Comput. Chem..

[20] Yan Y., Tao H., He J., Huang S.Y. (2020). The HDOCK server for integrated protein–protein docking. Nat. Protoc..

[21] Xue L.C., Rodrigues J.P., Kastritis P.L., Bonvin A.M., Vangone A. (2016). PRODIGY: A web server for predicting the binding affinity of protein–protein complexes. Bioinformatics.

[22] Rodrigues C.H.M., Pires D.E.V., Ascher D.B. (2021). DynaMut2: Assessing changes in stability and flexibility upon single and multiple point missense mutations. Protein Sci..

[23] Wolf J.M., Wolf L.M., Bello G.L., Maccari J.G., Nasi L.A. (2023). Molecular evolution of SARS-CoV-2 from December 2019 to August 2022. J. Med. Virol..

[24] Osman I.O., Levasseur A., Brechard L., Abdillahi Hassan I., Salah Abdillahi I., Ali Waberi Z., Delerce J., Bedotto M., Houhamdi L., Fournier P.-E. (2021). Whole Genome Sequencing of SARS-CoV-2 Strains in COVID-19 Patients From Djibouti Shows Novel Mutations and Clades Replacing over Time. Front. Med..

[25] Hodcroft E.B., Hadfield J., Neher R.A., Bedford T. (2020). Year-Letter Genetic Clade Naming for SARS-CoV-2 on Nextstrain.org.

[26] CoVariants (2020). Overview of Variants/Mutations.

[27] Lan J., Ge J., Yu J., Shan S., Zhou H., Fan S., Zhang Q., Shi X., Wang Q., Zhang L. (2020). Structure of the SARS-CoV-2 spike receptor-binding domain bound to the ACE2 receptor. Nature.

[28] Mahmood TBin Hossan M.I., Mahmud S., Shimu M.S.S., Alam M.J., Bhuyan M.M.R., Bin Emran T. (2022). Missense mutations in spike protein of SARS-CoV-2 delta variant contribute to the alteration in viral structure and interaction with hACE2 receptor. Immun. Inflamm. Dis..

[29] Alam M.M., Hannan S.B., Saikat T.A., Limon M.B.H., Topu M.R., Rana M.J., Salauddin A., Bosu S., Rahman M.Z. (2023). Beta, Delta, and Omicron, Deadliest Among SARS-CoV-2 Variants: A Computational Repurposing Approach. Evol. Bioinform..

[30] Zhang J., Cai Y., Lavine C.L., Peng H., Zhu H., Anand K., Tong P., Gautam A., Mayer M.L., Rits-Volloch S. (2022). Structural and functional impact by SARS-CoV-2 Omicron spike mutations. Cell. Rep..

[31] Moghaddar M., Radman R., Macreadie I. (2021). Severity, Pathogenicity and Transmissibility of Delta and Lambda Variants of SARS-CoV-2, Toxicity of Spike Protein and Possibilities for Future Prevention of COVID-19. Microorganisms.

[32] Bálint G., Vörös-Horváth B., Széchenyi A. (2022). Omicron: Increased transmissibility and decreased pathogenicity. Signal Transduct. Target. Ther..

[33] Zhu M., Zeng Q., Saputro B.I.L., Chew S.P., Chew I., Frendy H., Tan J.W., Li L. (2022). Tracking the molecular evolution and transmission patterns of SARS-CoV-2 lineage B.1.466.2 in Indonesia based on genomic surveillance data. Virol. J..

[34] Cahyani I., Putro E.W., Ridwanuloh A.M., Wibowo S., Hariyatun H., Syahputra G., Akbariani G., Utomo A.R., Ilyas M., Loose M. (2022). Genome Profiling of SARS-CoV-2 in Indonesia, ASEAN and the Neighbouring East Asian Countries: Features, Challenges and Achievements. Viruses.

[35] Harvey W.T., Carabelli A.M., Jackson B., Gupta R.K., Thomson E.C., Harrison E.M., Ludden C., Reeve R., Rambaut A., COVID-19 Genomics UK (COG-UK) (2021). SARS-CoV-2 variants, spike mutations and immune escape. Nat. Rev. Microbiol..

[36] Zhou W., Xu C., Wang P., Luo M., Xu Z., Cheng R., Jin X., Cuo Y., Xue G., Juan L. (2021). N439K Variant in Spike Protein Alter the Infection Efficiency and Antigenicity of SARS-CoV-2 Based on Molecular Dynamics Simulation. Front. Cell. Dev. Biol..

[37] Jhun H., Park H.-Y., Hisham Y., Song C.-S., Kim S. (2021). SARS-CoV-2 Delta (B.1.617.2) Variant: A Unique T478K Mutation in Receptor Binding Motif (RBM) of Spike Gene. Immune Netw..

[38] Wang C., Liu B., Zhang S., Huang N., Zhao T., Lu Q., Cui F. (2023). Differences in incidence and fatality of COVID-19 by SARS-CoV-2 Omicron variant versus Delta variant in relation to vaccine coverage: A world-wide review. J. Med. Virol..

[39] Barh D., Tiwari S., Rodrigues Gomes L.G., Ramalho Pinto C.H., Andrade B.S., Ahmad S., Aljabali A.A.A., Alzahrani K.J., Banjer H.J., Hassan S.S. (2023). SARS-CoV-2 Variants Show a Gradual Declining Pathogenicity and Pro-Inflammatory Cytokine Stimulation, an Increasing Antigenic and Anti-Inflammatory Cytokine Induction, and Rising Structural Protein Instability: A Minimal Number Genome-Based Approach. Inflammation.

[40] Rashid P.M.A., Salih G.F. (2022). Molecular and computational analysis of spike protein of newly emerged omicron variant in comparison to the delta variant of SARS-CoV-2 in Iraq. Mol. Biol. Rep..

[41] Nguyen H.L., Lan P.D., Thai N.Q., Nissley D.A., O’Brien E.P., Li M.S. (2020). Does SARS-CoV-2 Bind to Human ACE2 More Strongly Than Does SARS-CoV?. J. Phys. Chem. B.

[42] Bæk K.T., Mehra R., Kepp K.P. (2023). Stability and expression of SARS-CoV-2 spike-protein mutations. Mol. Cell. Biochem..

[43] Shuai H., Chan J.F.-W., Hu B., Chai Y., Yuen T.T.-T., Yin F., Huang X., Yoon C., Hu J.-C., Liu H. (2022). Attenuated replication and pathogenicity of SARS-CoV-2 B.1.1.529 Omicron. Nature.

[44] Upadhyay V., Lucas A., Panja S., Miyauchi R., Mallela K.M.G. (2021). Receptor binding, immune escape, and protein stability direct the natural selection of SARS-CoV-2 variants. J. Biol. Chem..

[45] Mehra R., Kepp K.P. (2022). Structure and Mutations of SARS-CoV-2 Spike Protein: A Focused Overview. ACS Infect. Dis..

