# Supplementary figures and images for: Dynamics of SARS-CoV-2 Spike RBD Protein Mutation and Pathogenicity Consequences in Indonesian Circulating Variants in 2020–2022
Source: Genes (Basel). 2024 Nov 14;15(11):1468. doi: 10.3390/genes15111468 (PMC11593803; doi:10.3390/genes15111468)

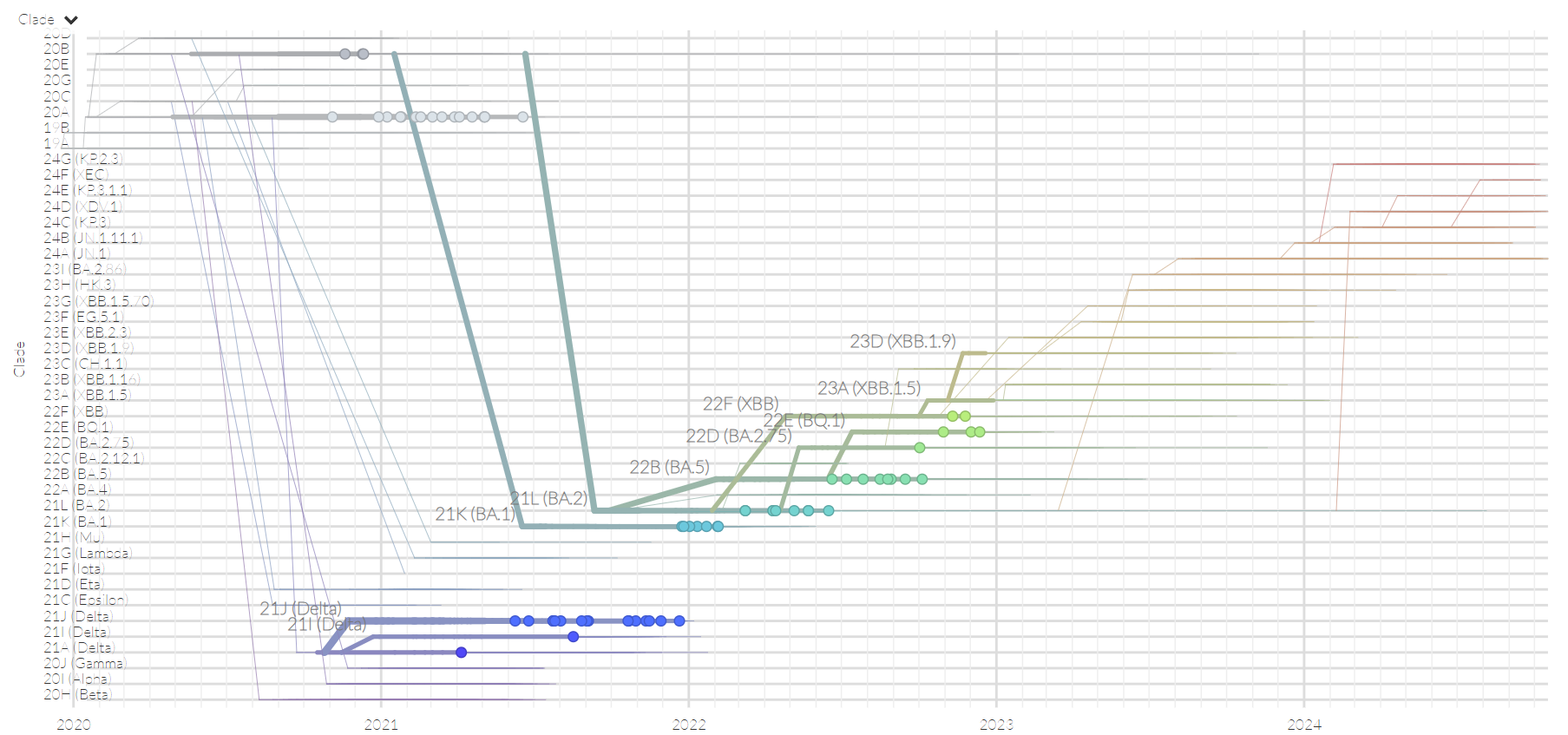

Supplement: Supplementary file 1 [file genes-15-01468-s001.zip › Supplementary figures S1.tiff]

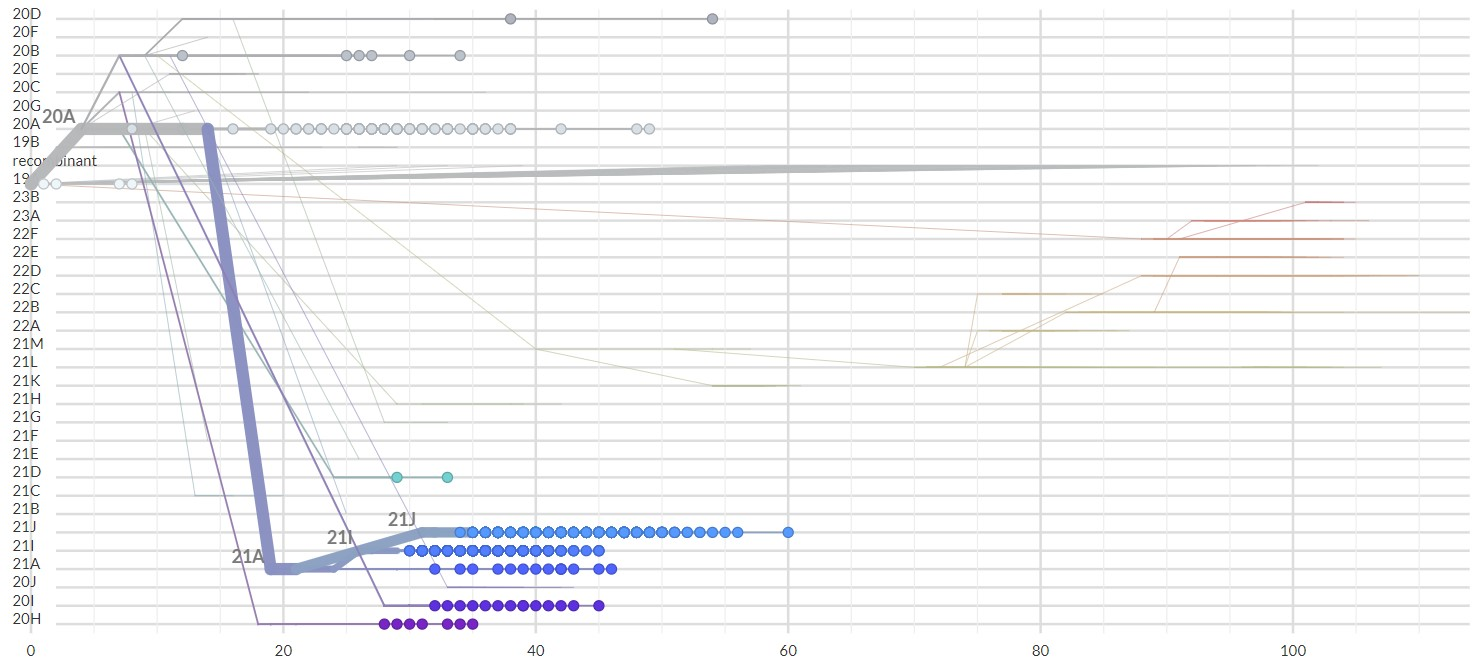

Supplement: Supplementary file 1 [file genes-15-01468-s001.zip › Supplementary figures S3.tiff]

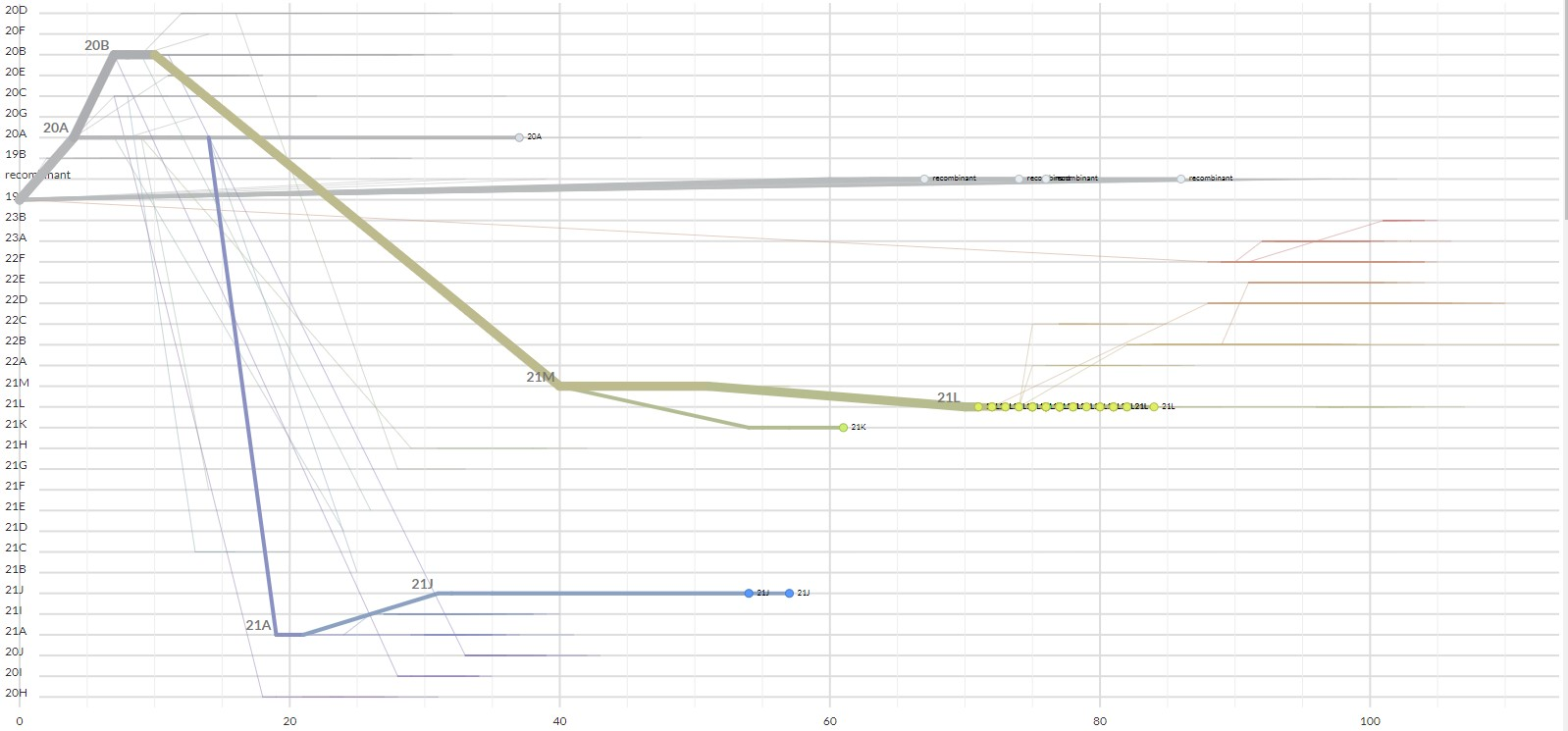

Supplement: Supplementary file 1 [file genes-15-01468-s001.zip › Supplementary figures S4.tiff]
